# Draft Genome Sequence of *Pasteurella multocida* subsp. *multocida* B:2 Strain VTCCBAA264 Isolated from *Bubalus bubalis* in North India

**DOI:** 10.1128/genomeA.00755-14

**Published:** 2014-07-31

**Authors:** R. K. Vaid, K. Shanmugasundaram, Ashok Boora, B. C. Bera, B. N. Shukla, Taruna Anand, Harisankar Singha, Thachamvally Riyesh, N. Virmani, S. Barua, V. B. Ahir, P. G. Koringa, M. R. Sajnani, Vaibhav D. Bhat, N. Rana, K. P. Singh, P. Malik, R. K. Singh, C. G. Joshi

**Affiliations:** a Veterinary Type Culture Collection, National Research Centre on Equines, Hisar, Haryana, India; b Central Institute for Research on Buffalo, Hisar, Haryana, India; c National Research Centre on Equines, Hisar, Haryana, India; d Department of Animal Biotechnology, College of Veterinary Science and Animal Husbandry, Anand Agricultural University, Anand, Gujarat, India

## Abstract

The *Pasteurella multocida* subsp. *multocida* B:2 serotype causes hemorrhagic septicemia in bubalines with high morbidity and mortality in the Indian subcontinent. We report the draft genome sequence of *Pasteurella multocida* strain VTCCBAA264 isolated from the small-intestine of a buffalo calf that died of high fever.

## GENOME ANNOUNCEMENT

North India is the cradle of high milk-yielding buffalo breeds which routinely suffer from haemorhhagic septicemia (HS), which has been estimated to cause huge economic losses (1). *Pasteurella multocida* subsp. *multocida*, normally an upper respiratory tract commensal, is responsible for HS outbreaks, which mostly occur in hot humid weather, indicating failure of immunity after vaccination or otherwise. The Asian serotype B:2 is mainly responsible for the disease in domestic ruminants (2). Although many of the molecular determinants for virulence of *P. multocida* are now identified, the pathogenesis of HS is still not well understood (3). The sequencing of this *P. multocida* strain VTCCBAA264, isolated from a buffalo calf, will be useful because of the wealth of its molecular clues.

This is the first whole-genome sequence of *Pasteurella multocida* isolate from north India. Strain VTCCBAA264, a B:2 serotype, was isolated from the morbid intestinal content of a buffalo (*Bubalus bubalis*) calf which died of high fever and respiratory distress on an organized buffalo dairy farm (4).

The genome sequencing was achieved by 454 pyrosequencing of a shotgun library. The sequence was assembled *de novo* using Newbler v2.6. A total of 123,415 reads were generated using the GS FLX Titanium system, giving ~23× coverage of the genome. The data generated 78 contigs ranging in size from 710 to 319,560 bp, with a total size of 2,280,332 bp, an *N*_50_ of 65,447 bp, and a Q40 of 99.65%. Annotation was carried out against strain Pm70 using the RAST server (5). The VTCCBAA264 strain showed a G+C content of 40.4% with 2,176 predicted genes, out of which there were 2,127 protein-encoding genes, 4 coded rRNAs, and 45 encoded tRNAs. Among the 2,127 coding sequences (CDSs), 2,074 (97.5%) were assigned to functional clusters of orthologous groups (COGs), 1,863 (87.6%) were assigned to FIGfams (fellowship for interpretation of genome families) and 1,527 (71.8%) to KEGG (Kyoto encyclopedia of genes and genomes). One clustered regularly interspaced short palindromic repeats (CRISPRs) array was also detected.

The cumulative sequence length of our genome compares favorably with the 2 other draft genome sequences of buffalo isolates of *P. multocida* B:2 serotype strains currently available, those for Anand1_buffalo (GenBank accession no. ALBY0100000000) and PMTB (AWTD01000000).

SEED subsystem analysis revealed various genes involved in a number of pathways (6). Noteworthy are the genes for antimicrobial resistance including translation elongation factor, DNA gyrase subunit A, topoisomerse IV subunit B, and DNA directed RNA polymerase β-subunit, among others. Genes for resistance to flouroquinolones and negative regulators of beta-lactamase expression were reported.

Copper is critical in the pathogenesis of bacterial pathogens (7). Analysis revealed copper-translocating chaperone, and copper homeostasis protein genes. The iron acquisition and hemin-transport system genes in this strain underlines its virulence potential. The genome also contained 45 phage related proteins. This whole-genome sequence and its functional analyses will be useful for realizing a universal vaccine for the prevention of HS.

### Nucleotide sequence accession numbers.

This whole-genome shotgun project has been deposited at DDBJ/EMBL/GenBank under the accession no. ALYC00000000. The version described in this paper is version ALYC02000000.
